# The Use of Dietary Interventions in Pediatric Patients

**DOI:** 10.3390/pharmacy7010010

**Published:** 2019-01-15

**Authors:** Shirin Madzhidova, Lusine Sedrakyan

**Affiliations:** 1 Department of Pharmacy Practice, Philadelphia College of Osteopathic Medicine, Georgia Campus, Suwanee, GA 30024, USA; 2 Byrd Dental Group, Alpharetta, GA 30022, USA; lusinesedrakyan91@gmail.com

**Keywords:** pediatric pharmacy, complementary alternative medicine, dietary interventions, oral manifestations, chronic pediatric conditions, ketogenic diet, gluten free casein free diet

## Abstract

Complementary and alternative treatment approaches are becoming more common among children with chronic conditions. The prevalence of CAM use among US adults was estimated to be around 42% in 2015 and around 44% to 50% among adults with neurologic disorders. Studies demonstrate that children with certain chronic illnesses such as asthma, cancer, genetic disorders, attention-deficit/hyperactivity disorder (ADHD), and other neurodevelopmental disorders are treated with complementary and alternative treatments at higher rates. Dietary therapies are gaining increasing popularity in the mainstream population. Although the majority of “fad” diets do not have enough supporting evidence, some dietary therapies have been utilized for decades and have numerous published studies. The objective of this review is to describe the dietary interventions used in children with the specific chronic conditions, to evaluate their efficacy based on published data and to encourage pharmacist involvement in the management and care of such patients.

## 1. Introduction

Complementary and alternative treatment approaches are becoming more common among children with chronic conditions. The National Center for Complementary and Alternative Medicine at the National Institute of Health (NIH) defines complementary and alternative medicine (CAM) as a “group of diverse medical and health care systems, practices, and products that are not presently considered to be part of conventional Western medicine” [1]. CAM treatments may be used in combination with traditional treatment approaches (complementary) or in place of traditional treatments (alternative), which is less common. There are various categories of CAM, such as biologically-based (e.g., herbs, vitamins, diets), manipulative and body-based (e.g., massage, chiropractic), mind–body (e.g., hypnosis, prayer), and biofield (e.g., acupuncture, homeopathy). The prevalence of CAM use among US adults was estimated to be around 42% in 2015 and around 44% to 50% among adults with neurologic disorders [2,3]. The NIH Center for Complementary and Integrative health estimated the use of CAM among US children to be at 11.6%, with natural products (fish oil/omega-3, melatonin, and probiotics) being the most commonly used [1]. Special diets accounted for 0.8% of CAM’s used and 2.2% of US children with conditions like ADHD used some form of CAM [1]. Other studies demonstrate that children with certain chronic conditions such as attention-deficit/hyperactivity disorder (ADHD) and other neurodevelopmental illnesses, utilize CAM at higher rates (24%) [4,5,6,7]. Among those, supplement and herbal medications, as well as dietary modifications (i.e., elimination or intake of specific foods) are most prevalently used at 31% and 17% respectively [7]. Many natural products, such as fish oil/omega-3 and probiotics, are well supported by studies for their place in the prevention and treatment of certain conditions in both adults and children. The various reported reasons for use of CAM by adults and caregivers of children include fear of adverse effects from conventional medications and perception of safety compared to traditional medicine. Parents of children with conditions that lack effective medical approaches or complete remissions often turn to alternative treatment approaches with the notion that they are generally risk-free. A survey of parents found that more than 50% had used at least on type of CAM therapy for their children with ASD, which is not always reported to the health-care provider [8,9]. Dietary therapies are gaining increasing popularity in the mainstream population, as families may seek advice from health practitioners such as naturopathic doctors, chiropractors and acupuncturists or lay advisers within their cultural communities [1]. Although the majority of “fad” diets do not have enough supporting evidence, some dietary therapies have been utilized for decades and have numerous published studies. Special diets, such as the ketogenic and the low-FODMAP diet, are commonly used as an alternative to conventional treatment, while most exclusion diets are utilized as a complement to their conventional treatment plans. Specific diets are discussed in detail below. Nevertheless, CAM approaches, such as dietary interventions, pose potential challenges when integrated with conventional treatments as well as with the risk of adverse effects. For those patients who are undergoing integrative treatment, close collaborative management from health-care providers is essential in ensuring the success of therapy and the well-being of the patient. 

The objective of this review is to describe the dietary interventions used in children with the specific chronic conditions, to evaluate their efficacy based on published data, and to encourage pharmacist involvement in the management and care of such patients.

## 2. Review of the Literature

**Figure 1:**
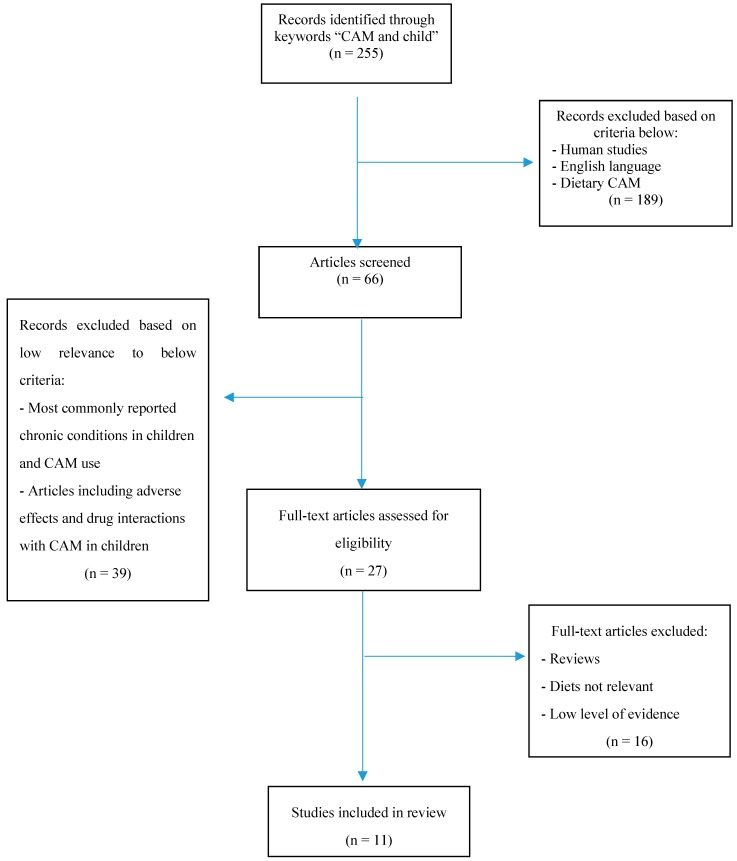
PRISMA Flow Diagram.

The literature review below was compiled using a systematic search of Medline database [10] from years 1975 to 2018. The search criteria included articles published in English language and restricted to human studies. Key words searched were combinations of complementary, alternative, diet, dietary, neurodevelopment, pediatric, naturopathy, homeopathy, IBS, Crohn’s, ADHD, ASD or chronic. Articles were selected based on the design, methodology and evaluation, and included randomized controlled trials (RCT), meta-analyses and review articles. Conditions and applicable diets were selected based on the availability of RCT’s and meta-analyses, as well as the applicability to the pharmacy profession. Figure 1 provides an overview of the literature selection process. The search identified a total of 255 publications with the inclusion of a combination of the above search terms. Following the removal of 189 irrelevant records, based on the established exclusion criteria, 66 publications remained. Of these, 39 were excluded as they did not meet the selection criteria, and 16 were removed based on its relevancy to the selected diets. As a result, a total of 11 studies are being included in this review.

## 3. Ketogenic Diet for Epilepsy

Epilepsy is a group of neurologic disorders characterized by episodes of recurring seizures, the cause of which is mostly unknown. Despite continued advancements in anticonvulsant pharmacotherapy, 30% of patients with epilepsy experience refractory seizures that are unresponsive to pharmacologic treatment or become intolerant to the side effects of medications [11]. The ketogenic diet (KD) is a non-pharmacologic treatment option for children with refractory seizures, which has been used worldwide for decades. The Ketogenic Diet Study Group, which is comprised of dieticians and other specialists in pediatrics, had published a consensus report outlining that the KD may be strongly considered in children with symptomatic generalized epilepsies who had failed two to three anticonvulsant therapies [12]. 

Fasting has been utilized since the 1920’s to alleviate symptoms of seizures, although the exact mechanism of action was not yet known at the time. It was believed that an intoxication of the brain from substances in the intestines was the main cause of epilepsy and fasting was reported to have high rates of efficacy [13]. It was later discovered that ketones were responsible for the anticonvulsant effect of fasting and can be produced in the absence of sufficient glucose through the oxidation of certain acids. Such acids are produced when the body consumes mainly fats as its primary energy source and therefore mimicking a state of fasting, thereby being termed the ketogenic diet. The ketogenic diet (KD) for the treatment of epilepsy was first reported in 1921 and has been studied extensively since [14]. The diet consists of mainly fat and protein consumption, with very low intake of carbohydrates (e.g., 4:1, 3:1, or 2:1 fat to non-fat ratio). Energy consumption mainly from fat is thought to mimic a state of ketosis. Fat metabolism through the liver leads to the production of three ketone bodies: beta-hydroxybutyrate (BHB), acetoacetate and acetone, which cross the blood-brain barrier and become the brain’s primary energy source [15]. 

Although not completely understood, several theories exist regarding the mechanism of action of the KD. It has been proposed that utilization of ketones for energy metabolism in the brain results in adaptive changes which increase energy reserves and gamma-aminobutyric acid (GABA) synthesis (major inhibitory neurotransmitter), resulting in seizure resistance [16]. Ketone bodies themselves are thought to possess anticonvulsant properties since they are structurally similar to GABA, BHB and acetoacetate. The diet has also been documented to be neuroprotective by inhibition of caspase-3-mediated apoptosis and through the activation of mitochondrial uncoupling proteins, which can reduce the production of reactive oxygen species [16]. 

The ketogenic diet encompasses various modalities of implementation, however, the majority of clinical data available are for the classic KD, which consists of 90% caloric intake from long-chain triglycerides and only 10% from carbohydrates and proteins, in a 4:1 ratio of fat to non-fat sources [16]. The classic KD is recommended for children, however, a 3:1 ratio for adolescents and a 2:1 ratio for infants may be used since more protein is required in these age groups [16]. Liquid KD formulas are available for bottle-fed infants and children with gastrostomy tube feedings. The diet is further modified to allow for appropriate growth and development of a child. Initiation of the KD most often takes place in an acute care setting, at an outpatient epilepsy center or as an inpatient at a hospital, in order to safely monitor ketone and glucose levels, with an average hospital stay of 4 days [17]. The diet is traditionally introduced slowly following a 24–48 h fasting period, until the patient fully tolerates the KD and is then discharged home. 

The efficacy of the KD on seizure activity in published studies varies, although the majority of studies show some reduction in seizure occurrence. A recent meta-analysis, which included 19 observational studies with a total of 1084 patients, found that close to 60% of patients had over a 50% seizure reduction, while 30% had over a 90% reduction in seizures, 6 months after initiation of the KD [17]. A randomized controlled trial including 145 children reported that the mean percentage of baseline seizures was significantly lower in the KD group at 3 months compared to the control group, which had experienced an increase in seizures from baseline [18]. 

Variations of the KD exist; although the most commonly utilized is the classic KD, the medium-chain triglyceride (MCT) diet, the modified MCT diet, the modified Atkins diet, and the low-glycemic index treatment diet can also be utilized. The MCT diet is comprised of mainly medium-chain fatty acids at 71% (as a specially formulated oil), 10% protein, and 19% carbohydrates [17]. The MCT is comprised of fat sources that produce more ketones than the long-chain triglycerides (LCT) used in the classic KD, therefore allowing for less fat consumption and more protein and carbohydrates to be incorporated into the diet [17]. Alternatively, the modified MCT diet combines the use of LCT (40–50% of calories) and MCT (30% of calories), as well as protein (10–20%) and carbohydrates (5–10%). A study comparing the MCT diet, classic KD, as well as a modification of both, reported that they were of approximately equal efficacy, with a higher incidence of gastrointestinal irritation with the MCT diet [17]. The modified Atkins diet (MAD) and low-glycemic-index (LGID) treatment diet both can utilize medium-chain or long-chain triglycerides (65% calories from fat), with a larger daily allowance of carbohydrate intake, which offers more flexibility in meal preparation to the caregiver [19]. These diets can be initiated in an outpatient setting. A study of 20 patients with retractable epilepsy on a modified Atkins diet showed greater than 50% reduction in seizures at 6 months in the majority of patients [20]. These results closely correlate to the efficacy of the classic KD. 

Although generally considered to be a safe treatment choice, KD has been shown to cause several adverse events in children and adults. During initiation of the diet, acidosis, dehydration, hypoglycemia, and gastrointestinal distress have been reported as the most prominent adverse events but are typically transient and easily managed [12]. Other reported adverse events associated with the KD maintenance include poor growth, nephrolithiasis, dyslipidemia, prolongation of QT interval, cardiomyopathy, excessive bruising, vitamin D deficiency, trace mineral deficiencies, constipation, and exacerbation of gastrointestinal reflux disease [12]. Cholesterol and lipids have been shown to be adversely affected, with a reported increase of total cholesterol of ~130%, which then stabilized over 2 years [12]. Certain conditions, such as the history of kidney stones, liver disease, dyslipidemia, gastroesophageal reflux disease, cardiomyopathy, constipation, or metabolic acidosis, may be aggravated by the diet and require close monitoring and testing [12]. 

Serious complications associated with the KD appear to be relatively rare, while the long-term complications are not well documented [21]. Overall, the KD can be an appropriate treatment option for epilepsy in children, and as with any other medical treatments, requires individualized care, close monitoring, and follow-up by the health-care provider. 

Pharmacists can play an important role in the management of patients on the KD and concomitant pharmacologic therapy. Many medications, specifically pediatric liquid preparations, have a high carbohydrate content, which may compromise ketosis. Anticonvulsants medications with the highest amounts of carbohydrates include carbamazepine suspension, phenobarbital elixir, ethosuximide syrup, and valproic acid syrup, and should be limited or avoided in children on the KD [22]. Alternatively, these patients may be given the capsule or crushed tablet formulation, which generally contains very low amounts of carbohydrates. For a summative list of common pediatric medications with high carbohydrate content refer to Table 1. Despite a long history of concomitant use of the KD and antiepileptic agents, evidence is insufficient to conclude what negative or positive drug interactions are of concern, and limited information exists regarding the effects of KD on pharmacokinetics of antiepileptics. Abnormal laboratory parameters may be seen in children on KD; however, metabolic acidosis requiring treatment may be more common with the concomitant use of topiramate or zonisamide, particularly at the initiation of KD [12]. Bicarbonate concentrations need to be closely monitored, particularly in the presence of concomitant anticonvulsant administration. Additionally, supplements containing bicarbonate should be reserved for patients who are clinically symptomatic (e.g., vomiting, lethargy) [12]. 

## 4. Gluten-Free Casein-Free Diet for Autism Spectrum Disorder

Prevalence of autism and autism spectrum disorder (ASD) has been on the rise and was most recently reported to occur in 1 in 59 US children [23]. According to the Diagnostic and Statistical Manual of Mental Disorders, Fifth Edition “autism is characterized by qualitative impairments in social interaction and communication, as well as restrictive, repetitive, and stereotyped patterns of behavior, interest and activity” [24]. The most recent updates to the manual include the combining of autism subtypes, such as autistic disorder and Asperger syndrome, into a unified diagnosis of autism spectrum disorder (ASD) [24,25]. 

Definitive etiology of ASD is not yet clearly understood since several studies attribute the disorder to genetic factors, metabolic derangements and environmental or dietary causes [26]. Gastrointestinal issues, such as chronic constipation or diarrhea, are among the most common medical conditions associated with autism, although a direct correlation has not been substantiated. A study comparing GI issues in children with autism and other neurodevelopmental illnesses (e.g., cerebral palsy) found that 70% of children with autism were affected compared with 42% of children with other neurodevelopmental illnesses and 28% of children with normal development [27]. In a study conducted by Campbell et al., 9% of unaffected siblings of children with ASD had a gastrointestinal disorder whereas the prevalence in children with autism was at 41% (*p* = 0.000) [28]. Considering the proposed etiology of GI involvement in ASD, many research articles have been published looking at dietary interventions to alleviate symptoms in children with ASD.

Specific dietary interventions in children with ASD include the omission of gluten and casein-containing foods [29]. The prevalence of use of the gluten-free and casein-free diet (GFCF) among children with ASD is estimated at 40% [30]. Gluten is a protein found in wheat, rye and barley, whereas casein is a protein found in dairy products [31]. The cessation of gluten and casein is based on the theory that opioid peptides may be produced due to a partial breakdown of foods that have gluten and casein. Such peptides can cross into the systemic circulation due to the increased intestinal permeability in children with ASD and further affect brain function and development [31]. Therefore, avoidance of foods containing gluten and casein is suggested to alleviate behavioral symptoms associated with ASD. Although widely reported and used, the diet and its proposed etiology lacks substantial evidence for efficacy, with only a few well-designed trials published. 

Although anecdotal reports from parents of success with the diet flood online forums, scientific evidence for its effectiveness remains inconclusive. A systematic review conducted in 2008 summarized two randomized controlled trials evaluating gluten-free casein-free (GFCF) diets in children with ASD [31]. While one of the studies concluded the GFCF diet significantly reduced the severity of symptoms of autism, the other study found no difference in the outcomes [31]. A recent randomized-controlled trial studied children with ASD who were assigned to either a GFCF diet or a low-sugar diet for 3 months in an open-label design [32]. No statistically significant differences were found between groups, although some behavioral and developmental benefits were observed in the GFCF group [32]. A study published in 2013 utilized a research synthesis technique to review major articles published on the use of GFCF diet in children with ASD [33]. In their assessment, the authors identified most studies that did not support the use of the GFCF diet in ASD and presented various limitations in the study design of the trials. Additionally, they noted most studies incorporated GFCF with other treatment modalities making it difficult to assess the effectiveness of GFCF alone, and subpopulations including Rett Syndrome and Childhood Disintegrative Disorder (CDD) require further studies to determine efficacy [33]. Overall, the American Academy of Pediatrics does not recommend the use of GFCF for ASD due to the lack of sufficient evidence, while the United Kingdom 2013 National Institute for Health and Care Excellence (NICE) clinical guideline on the management of ASD suggests that the potential risks of GFCF outweigh their benefits [34,35]. 

The majority of studies on GFCF did not report any serious adverse effects from the diet. However, an observational study on the provision of GFCF suggests casein restriction may lead to decreased bone mass and essential amino acid deficiency, such as tryptophan [36]. It is important for health-care providers to counsel families on the need for adequate vitamin D, calcium and protein supplementation, since most milk substitutes do not contain the appropriate amounts of protein. Another potential harm of adopting a GFCF diet is the potential to overlook possible underlying celiac disease or lactose intolerance [36]. Celiac disease is the most common autoimmune gastrointestinal disorder for which the treatment is complete avoidance of gluten [36]. Pharmacists should be aware of medications that may contain gluten as an excipient and be able to recommend alternative agents for patients with celiac disease or on a GFCF/gluten-free diet. For a list of common medication excipients containing gluten, as well as resources to find information regarding gluten-free medications, refer to Table 2 and Table 3. 

## 5. Specific Carbohydrate Diet (low FODMAPs) for Irritable Bowel Syndrome

Functional gastrointestinal disorder (FGID) is defined as a variable combination of chronic or recurrent gastrointestinal symptoms such as diarrhea, constipation and abdominal pain, which cannot be attributed to another medical condition [39]. Types of FGID include irritable bowel syndrome (IBS), functional abdominal pain, functional dyspepsia and abdominal migraine, with IBS being the most common [39]. The etiology of FGID is poorly understood, however, food intolerance such as poor absorption of carbohydrates has been implicated in the pathogenesis of FGID with recently emerging studies [40]. Most symptoms of irritable bowel syndrome (IBS) are due to luminal distension of the distal small and proximal large intestine, causing pain, bloating and abdominal distension, while some may also present with oral lesions, which are commonly missed upon diagnosis [41,42]. Solid, liquid or gas materials present in the gut can promote the distension of the lumen. Solids, mostly in the form of fiber, can either expand or contract the bacterial mass of the gut. Liquids may dictate the osmotic absorption or retention in the lumen, while gas can be ingested in the form of excess nitrogen, but is mostly produced by bacterial fermentation [42]. Therefore, dietary components that may lead to these changes in the lumen of the intestine are generally poorly absorbed, are small molecules, and can be readily fermented by bacteria. 

Fermentable Oligo-, Di- and Monosaccharides and Polyols (FODMAPs), are short-chain carbohydrates and sugar alcohols (polyols) which comprise fructose, lactose, fructo- and galactooligosaccharides (i.e., fructans, galactans), and polyols (e.g., sorbitol, mannitol, xylitol, maltitol) [42]. These dietary components have three common properties: they are poorly absorbed in the small intestine, they are small and osmotically-active molecules, and they are rapidly fermented by bacteria. For a list of High-FODMAP food sources, refer to Table 4. All of these properties can potentially contribute to the exacerbation of FGID symptoms. Thus, a low-FODMAP diet can improve gastrointestinal symptoms by reducing the amount of undigested carbohydrates in the presence of colonic bacteria, leading to less fermentation, which can lead to decreased abdominal bloating and pain as well as flatulence [43]. 

Numerous studies in adults have demonstrated significant improvement of IBS symptoms in patients on the low FODMAP diet, showing significantly higher satisfaction with stool consistency and decreased abdominal pain, bloating and flatulence [44]. Limited studies exist, however, for the use of the low FODMAP diet in children with IBS. Two studies, with limited power, studied the effects of fructose on the GI tract and elimination of fructose in children with fructose malabsorption [45,46]. The studies indicated that the administration of fructose produced a positive hydrogen breath test in 11 out of 32 children and fructose elimination was effective in reducing functional abdominal pain symptoms in 77% of studied children [45,46]. A double-blind randomized controlled trial of 54 children with IBS looked at a low-FODMAP diet compared to a high-FODMAP diet using a crossover design [47]. The authors found fewer episodes of abdominal pain, less nausea, less bloating and lower breath hydrogen production after 2 days on the diet [47]. Further studies in children are warranted to confirm the efficacy of the low-FODMAP diet for IBS and to determine its value in other forms of FGID. 

Although limited, reports regarding the safety of the low-FODMAP diet indicate that certain risks exist. Due to the limited ingestion of foods that are considered prebiotics, the gut microflora may be diminished, which could potentially be detrimental to large bowel health (e.g., promotion of colorectal carcinogenesis) [48]. The lack of fiber intake could arise from restricted intake of wheat-containing foods. In adolescents, the possibility of eating disorders comes into play, as a result of the innate possibility of IBS or food restrictions with the diet [48]. Close monitoring and counseling by a dietician is essential to ensure compliance and positive outcomes with the diet. For the patients who are on the diet, it is important for pharmacists to consider the presence of fructose or lactose in some pediatric drug formulations that may potentially worsen symptoms as well as counsel on the importance of vitamin and mineral supplementation.

## 6. Dietary Interventions for ADHD

Attention deficit hyperactivity disorder (ADHD) is a common neurodevelopmental condition present among school-age children. According to the American Psychiatric Association’s Diagnostic and Statistical Manual, Fifth edition (DSM-5), ADHD is distinguished by symptoms of inattention, overactivity, and/or impulsiveness which are age inappropriate, persistent, and pervasive [50,51]. ADHD presents with a probable future risk of school difficulties, communication challenges, mental illness and delinquency, which puts a significant load on families, as well as on the social and health care systems [52]. 

Generally, pharmacologic treatments for management of ADHD are preferred and widely used; however, a multimodal approach to treatment is recommended. A variety of non-pharmacologic and dietary interventions for the management of ADHD have been studied with mixed results. One of the earliest studied dietary interventions for ADHD is the Feingold diet, which was introduced in the 1970’s by Dr. Feingold who believed certain additives in food were associated with hyperactivity [53]. Foods avoided on the Feingold diet include processed meats, apples, grapes, and drinks with artificial flavors and coloring agents [53]. Products containing red and orange synthetic dyes, as well as preservatives like butylated hydroxytoluene and butylated hydroxyanisole are advised against [53]. The diet gained popularity when initially introduced to physicians and was claimed to ameliorate symptoms in more than 50% of children treated for hyperactivity [54]. Several controlled studies performed since failed to show the same efficacy; however, a small subgroup of children that may be susceptible have been identified [54]. More recent versions of the diet recommend avoiding artificial food coloring and additives only [55]. 

A meta-analysis published in 2012 evaluated studies on restriction diets for ADHD, in particular, elimination of artificial food coloring [56]. From the 34 high-quality studies selected, the authors published that while parent reports yielded a statistically significant reduction in symptoms among patients who eliminated food dyes, teacher/observer reports yielded no significant effect [56]. This illustrates the concept of observer bias, since parents are more likely to think an intervention is helping their child, therefore influencing the results. The authors concluded that an estimated 8% of children with ADHD may have symptoms related to synthetic food dyes and that further studies are warranted [56]. 

Another commonly used dietary intervention for children with ADHD is an oligoantigenic (hypoallergenic/elimination) diet. Oligoantigenic diet eliminates most known sensitizing food antigens or allergens, such as cow’s milk, cheese, wheat cereals, egg, chocolate, nuts, and citrus fruit, in an effort to identify and manage food allergies and intolerances that may be linked to neurologic dysfunction [57]. More recently known as an “elimination diet”, these diets may vary in their specific contents. A multi-food elimination diet, such as the 6-food elimination diet, excludes most food allergens. Alternatively, a “few foods diet” consists of consuming only foods with low antigenic potential, such as lamb/venison, quinoa/rice, pear, and a few others [58]. Individuals on a “few foods diet” must be closely monitored by a dietician to avoid nutritional deficiencies. Most elimination diets follow a two-step process, where foods are excluded for a period of time, after which foods are reintroduced one at a time to identify those that are causing symptoms [59]. 

Two recent meta-analyses were conducted to evaluate the diet effects of both restriction/elimination diets and food coloring agents on symptoms of ADHD. The authors concluded that the diet effect on children with ADHD, especially those with severe symptoms, can be higher than in those without ADHD, and that elimination diets might work. However, both meta-analyses noted the questionable study methods in most evaluated studies, as well as the difficulty in generalizing symptom improvement [56,60]. 

Overall, data on the effectiveness of elimination diets are conflicting and require additional, well-designed studies with a large sample size. For those parents of children with ADHD who do choose to implement elimination diets in their treatment regimen, pharmacists are able to assist with the proper selection of medication excipients. Many liquid pediatric formulations contain food dyes, artificial sweeteners, as well as allergens. By identifying the origin of the excipient in the prescribed or over-the-counter medications, pharmacists can help patients avoid those triggers and maintain their diet regimen. While many over-the-counter pediatric formulations are available as a dye-free alternative, pharmacists can be a great resource to locate information regarding food dyes and sweeteners used for prescription medications and flavoring agents. Although many prescription medications may list dyes and sweeteners in the excipient list, some dyes may be present in binding agents, which may not be found in the package insert. In the instance of absent information regarding dyes, it is recommended for the pharmacist to contact the manufacturer directly. 

## 7. Conclusions

Neurodevelopmental disorders are complex in nature, and their pathophysiology is not yet completely understood. Due to the challenges with the selection of the appropriate pharmacologic management, complementary and alternative treatment modalities are becoming more common among pediatric patients. Many parents feel that dietary interventions are a safe alternative, especially in the cases of conventional treatment failure. Although generally considered safe, dietary interventions do pose certain risks and require proper management. Pharmacists can play an important role in education of pediatric patients and their parents on the safety and efficacy of various CAM therapies as well as in managing their diets and preventing adverse effects (Appendix A). Communication with patients continues to prove its importance in many facets of pharmacotherapeutic management, but is ever more valuable for those patients also utilizing complementary and alternative therapies. The majority of the dietary interventions mentioned in this article do not have enough evidence to support use as monotherapy. Therefore, larger and better-structured studies are necessary to further identify their place in the management of chronic disorders in children.

## Figures and Tables

**Table 1:** Common pediatric medications with high carbohydrate content (≥2 grams/dose) [22] *.

	Dosage Unit
Acetaminophen liquid suspension (cherry) (Tylenol)	160 mg/5 mL
Acetaminophen elixir with codeine (Tylenol with Codeine) × 0.35 g ethyl alcohol/5 mL	120 mg/5 mL
Amoxicillin oral suspension (Trimox)	125 mg/5 mL
Ampicillin oral suspension (Omnipen)	125 mg/5 mL
Carbamazepine suspension (TEGretol)	100 mg/5 mL
Cephalexin oral suspension (Keflex)	125 mg/5 mL
Phenobarbital elixir ×0.71 g ethyl alcohol/5 mL	20 mg/5 mL
Valproic acid syrup (Depakene)	250 mg/5 mL

* For a more comprehensive list of medications refer to article reference [22].

**Table 2:** Common Medication Excipients that May Contain Gluten [37,38].

Excipient	Gluten-Free Botanical Source	Gluten Containing Botanical Source
Starch	Corn, potato, tapioca	Wheat
Pregelatinized starch, pregelatinized modified starch, sodium starch glycolate	Corn, rice, potato	Wheat
Dextrans	Corn, potato	Wheat, barley
Dextrose	Corn	Wheat, barley
Dextrates, dextrins	Corn, potato	Wheat, barley
Maltodextrin	Corn, potato	Wheat, barley
Caramel coloring	Corn	Barley malt

**Table 3:** Resources for more information about gluten in medications.

List of medications verified to be gluten-free	www.glutenfreedrugs.com
“A guide through the Medicine Cabinet” (book)	In print
Walgreens and CVS pharmacy OTC brand medication list	Available upon request
Additional information on gluten in foods and products	www.celiac.org www.celiaccentral.org

**Table 4:** High-FODMAP Carbohydrate Food Sources (to be avoided) [49].

Fructo-oligosaccharides (fructans)	Wheat, rye, onions, garlic, artichokes
Galacto-oligosaccharides (GOS)	Legumes
Lactose	Milk and milk products
Fructose	Honey, apples, pears, watermelon, mango
Sorbitol	Apples, pears, stone fruits, sugar-free mints/gums
Mannitol	Mushrooms, cauliflower, sugar-free mints/gums

## Appendix A

**Table A1:** Overview of the characteristics of the respective dietary interventions.

Diet	Diet Modality	Target Condition	Evidence	Potential Adverse Effects	Pharmacist Considerations
Ketogenic Classic MCT Modified Atkins LGI	Calories from 90% fat and 10% carbohydrate and proteins Medium-chain triglycerides Long-chain triglycerides	Refractory Epilepsy	RCT’s and meta-analyses available show statistically significant positive results [16,17]	Vomiting, diarrhea, acidosis, dehydration, hypoglycemia, poor growth, kidney stones, vitamin deficiency, etc.	Monitor carbohydrate content in liquid formulations; bicarbonate levels; concomitant anticonvulsants
Gluten-free Casein-free	Exclusion of gluten and casein (e.g., products containing wheat, oats, barley, or rye; and milk and dairy products)	ASD	Results are inconclusive with weak evidence for positive effects. A subset of patient with GI disturbances may benefit [30,31,32,33,34]	Potential decrease in bone mass; deficiencies in essential amino acids, vitamin D, calcium, and protein	Counseling on vitamin supplementation; availability of gluten-free medications (both OTC and Rx)
Low FODMAPs	Exclusion of short-chain carbohydrates (wheat, rye, garlic, legumes, etc.) and sugar alcohols (mannitol, sorbitol, etc.)	IBS	Results are inconclusive with weak evidence for positive effects. Diet did show improvement in symptoms such as bloating, nausea, and abdominal pain. [39,40]	Diminished microflora; lack of fiber intake leading to constipation; possible effect on growth; eating disorders	Counseling on vitamin supplementation; monitor for presence of high-FODMAP excipients in medications (both OTC and Rx)
Feingold Oligoantigenic	Elimination of artificial food coloring, flavors, fragrances, preservatives and sweetners. Elimination of antigenic foods (diet restricted to hypoallergenic foods: turkey, pears, rice, lettuce, water)	ADHD	Meta-analyses showed weak or no evidence for positive effects. Children with severe ADHD may experience larger effects on symptom resolution. [58,59,60]	Possible vitamin, mineral, fiber and protein deficiencies	Counseling on vitamin supplementation; selection of medications without artificial dyes or sweeteners.
